# Retraction Note: Protective functions of myricetin in LPS-induced cardiomyocytes H9c2 cells injury by regulation of MALAT1

**DOI:** 10.1186/s40001-024-01807-6

**Published:** 2024-04-01

**Authors:** Jinliang Sun, Jianhui Sun, Xuezhong Zhou

**Affiliations:** Department of Cardiology, The First People’s Hospital of Changzhou, No. 185 Juqian Street, Changzhou, 213000 China

**Retraction to: Eur J Med Res (2019) 24:20** 10.1186/s40001-019-0378-5

The Editors-in-Chief have retracted this article following an investigation by the National Health Commission of the People's Republic of China. The investigation found the involvement of a third party in writing and submission of the article. Additionally, concerns were raised for similar background in western blot images and similar characteristic layouts in bar graphs with other articles published around the same time. Therefore, the Editors-in-Chief no longer have confidence in the integrity of the data in this article.

None of the authors have responded to any correspondence from the editor/publisher about this retraction.

